# Supplementary material for: Multidrug-Resistant Helicobacter pylori Strains: A Five-Year Surveillance Study and Its Genome Characteristics
Source: Antibiotics (Basel). 2022 Oct 11;11(10):1391. doi: 10.3390/antibiotics11101391 (PMC9598532; doi:10.3390/antibiotics11101391)
Supplement: Supplementary file 1 [file antibiotics-11-01391-s001.zip › antibiotics-1956875-supplementary.pdf]

Table S1: Quality of genome sequencing

Assembly	HP18045	HP21041
# contigs (>= 0 bp)	34	41
# contigs (>= 1000 bp)	30	38
# contigs (>= 5000 bp)	21	26
# contigs (>= 10000 bp)	18	22
# contigs (>= 25000 bp)	16	20
# contigs (>= 50000 bp)	12	11
Total length (>= 0 bp)	1631102	1614323
Total length (>= 1000 bp)	1628188	1612108
Total length (>= 5000 bp)	1606665	1579305
Total length (>= 10000 bp)	1584197	1544131
Total length (>= 25000 bp)	1548405	1507772
Total length (>= 50000 bp)	1410211	1172137
# contigs	34	41
Largest contig	309238	176430
Total length	1631102	1614323
GC (%)	38.81	39.21
N50	133035	93545
N75	69574	47309
L50	4	7
L75	9	12
# N's per 100 kbp	0	0

Table S2: List of antibiotic resistance genes predicted to occur in HP18045 using RGI bioinformatic tool.

Best Hit ARO	Best Identities	ARO	Model_type	SNPs in Best Hit ARO	Other SNPs	Antibiotic Class	Resistance Mechanism	AMR Gene Family
vanT gene in *vanG* cluster	33.24	3002972	protein homolog model	n/a	n/a	glycopeptide	antibiotic target alteration	glycopeptide resistance gene cluster; *vanT*
Helicobacter pylori pbp2 mutants conferring resistance to amoxicillin	98.3	3007058	protein variant model	S494H, E572G	n/a	cephalosporin; cephamycin; carbapenems	antibiotic target alteration	Penicillin-binding protein mutations conferring resistance to beta-lactam antibiotics
Helicobacter pylori rpoB mutation conferring resistance to rifampicin	99.1	3007051	protein variant model	I837V, K2068R	n/a	fluoroquinolone; rifamycin	antibiotic target alteration; antibiotic target replacement	rifamycin-resistant beta-subunit of RNA polymerase *(rpoB)*
Helicobacter pylori frxA mutation conferring resistance to metronidazole	100	3007059	protein variant model	Y62D	n/a	nitroimidazole	antibiotic target alteration	Antibiotic resistant *Helicobacter pylori* nitroreductase

Helicobacter pylori pbp1 mutants conferring resistance to amoxicillin	97.42	3007060	protein variant model	V374L, S595G	n/a	cephalosporin; cephamycin; carbapenems	antibiotic target alteration	Penicillin-binding protein mutations conferring resistance to beta-lactam antibiotics
Helicobacter pylori rdxA mutation conferring resistance to metronidazole	94.76	3007055	protein variant model	R90K, A118S, C49T, D59N	n/a	nitroimidazole	antibiotic target alteration	Antibiotic resistant *Helicobacter pylori* nitroreductase
Helicobacter pylori 23S rRNA with mutation conferring resistance to clarithromycin	99.26	3004134	rRNA gene variant model	C1707T, A2144G	n/a	macrolide	antibiotic target alteration	23S rRNA with mutation conferring resistance to macrolide antibiotics

ARO:antibiotic resistance orthologoue; SNP: single nucleotide polymorphism; AMR: antimicrobial resistance

Table S3: List of antibiotic resistance genes predicted to occur in HP21041 using RGI bioinformatic tool.

Best Hit ARO	Best Identities	ARO	Model type	SNPs in Best Hit ARO	Other SNPs	Antibiotic Class	Resistance Mechanism	AMR Gene Family
vanTr gene in *vanL* cluster	35.99	3002974	protein homolog model	n/a	n/a	glycopeptide	antibiotic target alteration	glycopeptide resistance gene cluster; *vanT*
Helicobacter pylori pbp2 mutants conferring resistance to amoxicillin	96.94	3007058	protein variant model	S494H, E572G	n/a	cephalosporin; cephamycin; carbapenems	antibiotic target alteration	Penicillin-binding protein mutations conferring resistance to beta-lactam antibiotics
Helicobacter pylori pbp1 mutants conferring resistance to amoxicillin	96.51	3007060	protein variant model	T593A, G595S	n/a	cephalosporin; cephamycin; carbapenems	antibiotic target alteration	Penicillin-binding protein mutations conferring resistance to beta-lactam antibiotics
Helicobacter pylori gyrA conferring resistance to fluoroquinolones	95.65	3007052	protein variant model	T87I, N87I	n/a	fluoroquinolone	antibiotic target alteration	fluoroquinolone resistant *gyrA*

Helicobacter pylori rpoB mutation conferring resistance to rifampicin	98.65	3007051	protein variant model	K2068R, Q2079K	n/a	fluoroquinolone; rifamycin	antibiotic target alteration; antibiotic target replacement	rifamycin-resistant beta-subunit of RNA polymerase (*rpoB*)
Helicobacter pylori 23S rRNA with mutation conferring resistance to clarithromycin	99.13	3004134	rRNA gene variant model	A2147G, C1707T, A2144G	n/a	macrolide	antibiotic target alteration	23S rRNA with mutation conferring resistance to macrolide antibiotics

ARO:antibiotic resistance orthologoue; SNP: single nucleotide polymorphism; AMR: antimicrobial resistance.

<u>Figure S1: Distribution of minimum inhibitory concentration (MIC) of the tested antibiotics</u>

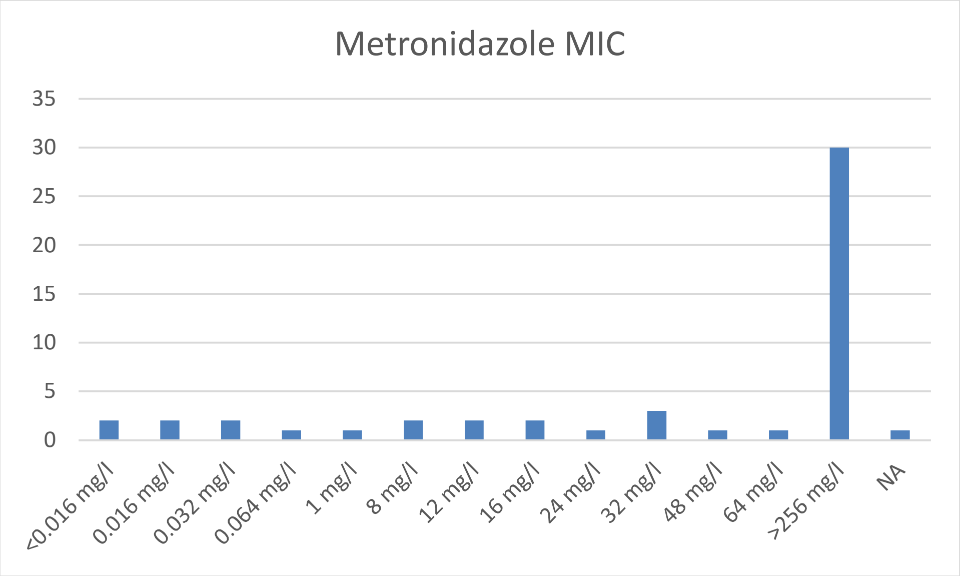

Figure S1a: Distribution of MIC in metronidazole. x-axis is MIC value while y-axis is number of *H. pylori* isolates

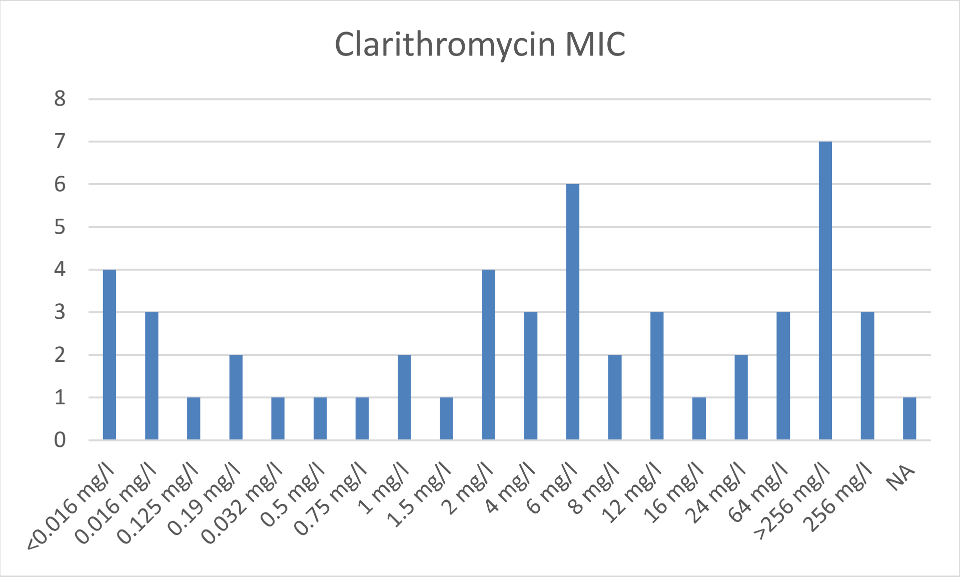

Figure S1b: Distribution of MIC in clarithromycin. x-axis is MIC value while y-axis is number of *H. pylori* isolates

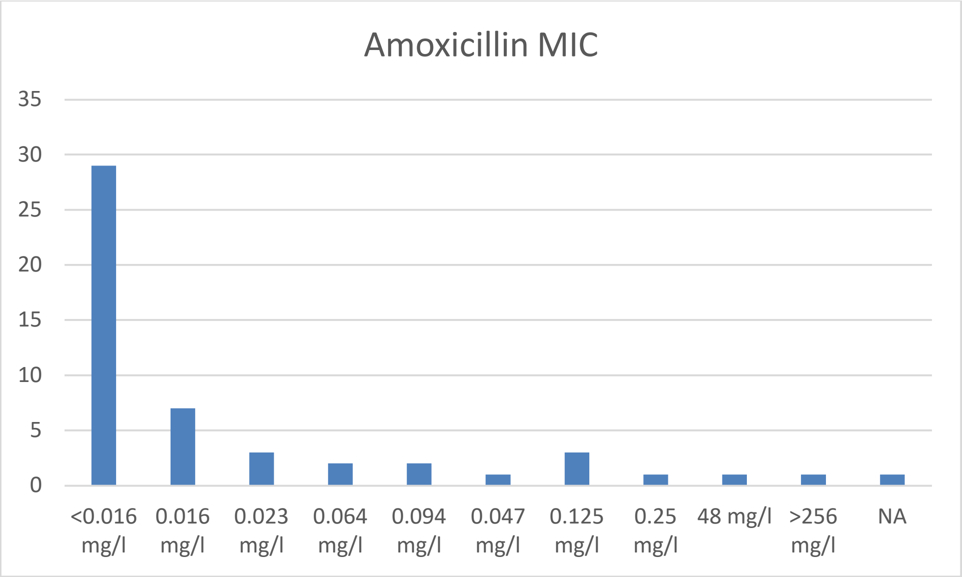

Figure S1c: Distribution of MIC in amoxicillin. x-axis is MIC value while y-axis is number of *H. pylori* isolates

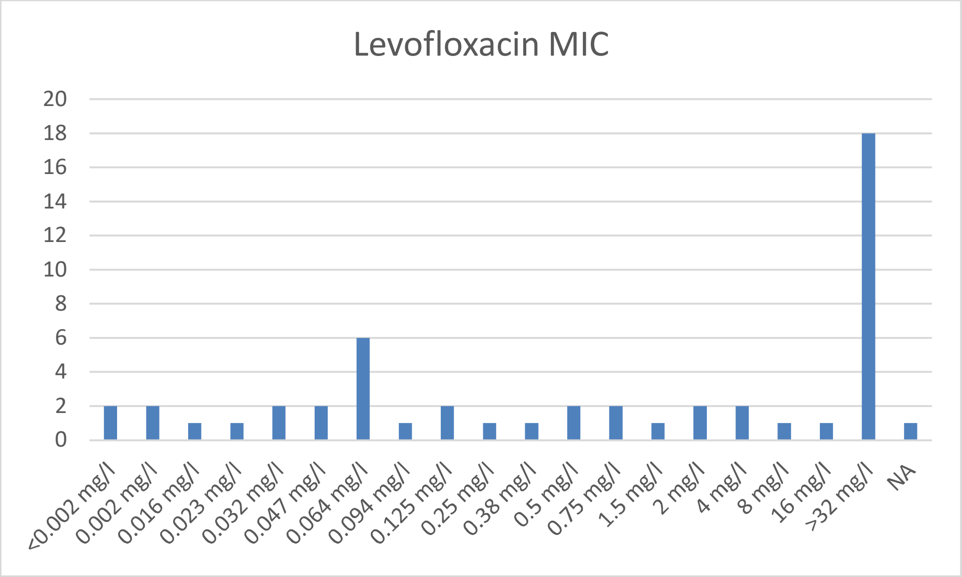

Figure S1d: Distribution of MIC in levofloxacin. x-axis is MIC value while y-axis is number of *H. pylori* isolates
